# High-risk adenoma predicts delayed post-polypectomy bleeding: a size-stratified retrospective cohort study and risk score development

**DOI:** 10.3389/fmed.2026.1913120

**Published:** 2026-08-31

**Authors:** Yiming Ding, Huihui Li, Xiangchun Lin

**Affiliations:** Department of Gastroenterology, Peking University International Hospital, Beijing, China

**Keywords:** delayed post-polypectomy bleeding, endoscopic mucosal resection, high-risk adenoma, interaction, risk score, size-stratified analysis

## Abstract

**Background:**

Delayed post-polypectomy bleeding (DPPB) is a common adverse event after endoscopic mucosal resection (EMR) of colorectal polyps. However, the role of high-risk adenoma (HRA) in DPPB has been rarely studied, as prior work focused on polyp number, age, and comorbidities; its size-specific effect remains unclear.

**Methods:**

Conducted at a single center, this retrospective cohort study enrolled 4,035 patients undergoing EMR for colorectal polyps (October 2016–March 2025) after excluding inflammatory bowel disease, familial polyposis, incomplete records, and non-EMR techniques. The HRA–DPPB association was evaluated with sequential multivariable logistic regression across three nested adjustment models, with interactions tested against high-risk serrated polyp (HRSP) and polyp size (≤1 cm vs. >1 cm). A simplified clinical risk score derived by the Sullivan method was internally validated through an optimism bootstrap that rebuilt the score on every resample, assessing discrimination (area under the curve, AUC) and calibration.

**Results:**

Of 4,035 patients, 106 (2.63%) developed DPPB. HRA was independently associated with DPPB (adjusted odds ratio [OR] 2.90, 95% confidence interval [CI] 1.74–4.85), and the estimate strengthened rather than weakened with sequential adjustment (crude OR 2.49). Pathology-stratified analysis showed a dose–response pattern—HRA only (OR 2.90), HRSP only (OR 2.27), both (OR 5.54)—with an additive interaction that trended positive without reaching significance [relative excess risk due to interaction (RERI) 1.37, 95% CI −2.65 to 5.38]. The HRA × polyp size interaction (*P* = 0.005) was driven by the overlap between the HRA size criterion (≥1 cm) and the size-based grouping: when HRA was redefined by its non-size criteria alone (high-grade dysplasia, villous component, or ≥3 adenomas), the interaction disappeared (*P* = 0.95), leaving histology (OR ≈ 1.5–2.1) and size (OR 7.4 at 2 cm vs. 0.8 cm) as independent predictors of bleeding. The simplified score [age, sex, polyp size, count, body mass index (BMI)] achieved an optimism-corrected AUC of 0.729 with adequate calibration (Hosmer–Lemeshow *P* = 0.222; corrected slope 0.923), stratifying patients into low (0.73%), medium (2.03%), and high (8.84%) risk groups.

**Conclusion:**

HRA is an independent risk factor for DPPB after EMR, and high-risk histology predicts bleeding across the size spectrum, complementing a strong, independent effect of polyp size. A simplified clinical score stratifies risk adequately, but as a single-center, internally validated instrument it awaits external confirmation before clinical adoption.

## 1 Introduction

Colorectal cancer (CRC) ranks third in global incidence and second in cancer-related mortality (1). Colorectal adenomas progress toward carcinoma through the conventional adenoma–carcinoma sequence, whereas roughly 15%–30% of CRCs arise from serrated polyps (2, 3). Endoscopic mucosal resection (EMR) is now the mainstay of colorectal polyp treatment, offering minimal trauma and rapid recovery, yet it carries complications such as bleeding, perforation, and post polypectomy coagulation syndrome (4, 5). Among these, delayed post-polypectomy bleeding (DPPB) is the most frequent, occurring in 6%–7% of large lesions (6). Defined conventionally as hemorrhage within 30 days of polypectomy, DPPB prolongs hospital stay, increases cost, and may prove life-threatening in severe cases (7). Identifying its risk factors early and strengthening the care of susceptible patients are therefore of clear clinical value.

Earlier studies have linked DPPB to several factors, including polyp size, location, and anticoagulant use (8). Whether high-risk adenoma (HRA) independently predisposes to DPPB, however, has received little attention (9). HRA is defined by a diameter ≥10 mm, high-grade dysplasia, a villous component, or the presence of three or more adenomas; because size is embedded in this definition, polyp size may confound the HRA–bleeding association or modify its effect (10). Beyond size, HRA also captures villous architecture, high-grade dysplasia, and overall adenoma burden—features with distinct vascular and architectural properties that may bear on bleeding independently of lesion size. Evidence on the HRA–DPPB relationship remains scarce, and no study has yet examined how HRA interacts with polyp size in driving DPPB.

A simple, reliable tool for predicting DPPB is also lacking. Nomogram-based models have been proposed for risk assessment, yet their bedside application remains cumbersome (11). A simplified risk score can fill this gap by translating a complex regression model into an integer point table that clinicians can tally quickly to gauge post-polypectomy bleeding risk.

This study therefore set out to (1) determine whether HRA is associated with DPPB; (2) probe the HRA–polyp size interaction through pathology- and size-stratified analyses; and (3) derive a simplified clinical risk score from the identified predictors and evaluate its discrimination and calibration using bootstrap internal validation.

## 2 Materials and methods

### 2.1 Study design and population

**Figure 1:**
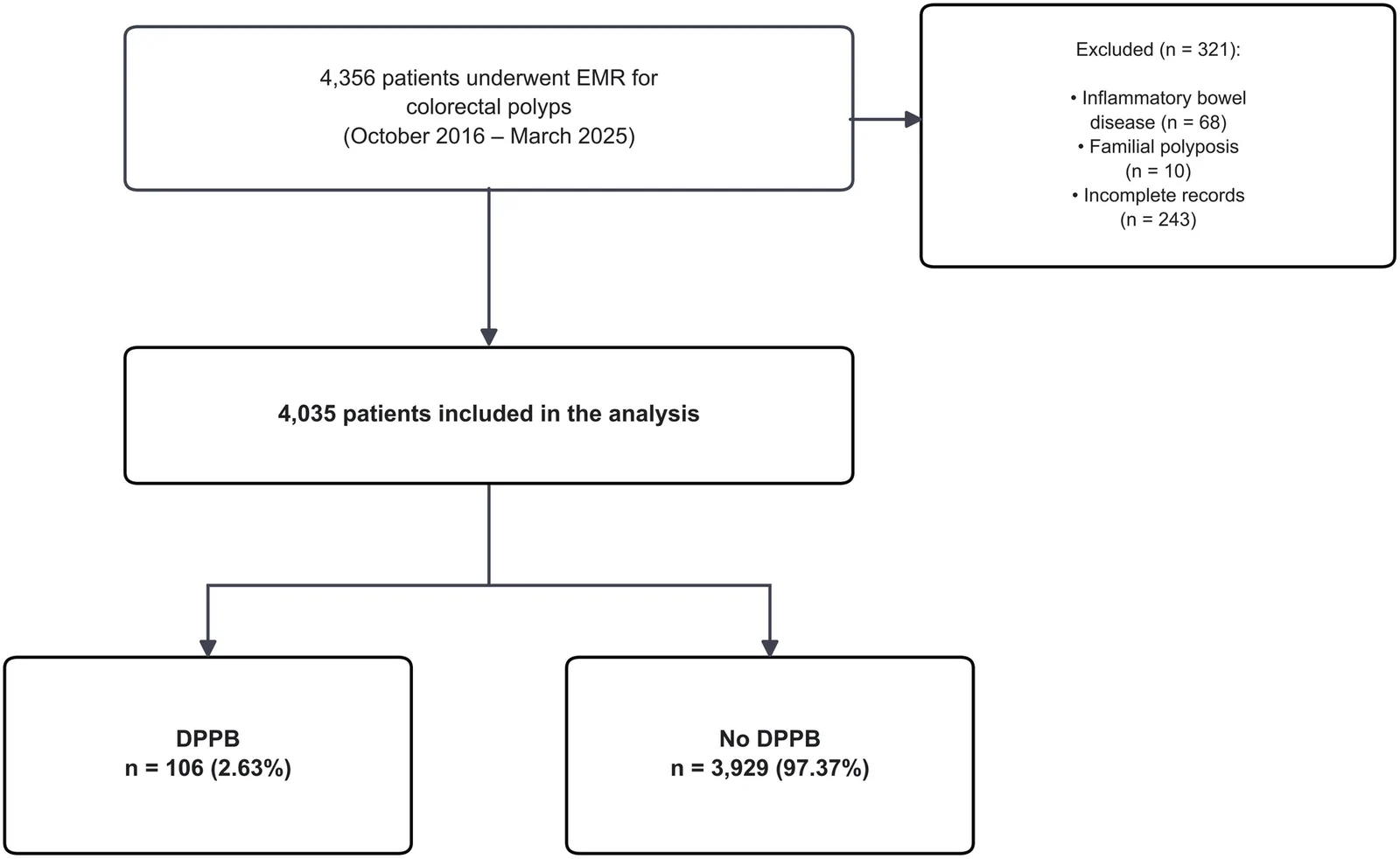
Patient flow diagram.

In this retrospective cohort study, we enrolled consecutive patients who underwent EMR for colorectal polyps at Peking University International Hospital between 1 October 2016 and 31 March 2025. The inclusion criteria were as follows: (1) age 18–85 years; (2) endoscopically confirmed colorectal polyps; (3) signed procedural informed consent; and (4) absence of contraindications to endoscopic therapy. The exclusion criteria were inflammatory bowel disease, familial adenomatous polyposis, incomplete clinical or pathological records, and treatment by endoscopic submucosal dissection or cold snare polypectomy, which preserves a homogeneous EMR cohort; the numbers excluded under each criterion are reported in the Results (Section 3.1, Figure 1). Each record was verified manually for resection technique.

The Peking University International Hospital Ethics Committee approved the protocol on October 24, 2025 (approval 2025-KY-0101-01) and waived informed consent given the retrospective design.

### 2.2 Data collection and variable definitions

We collected data across three domains. Baseline characteristics comprised age, sex, BMI, hypertension, diabetes, coronary heart disease, history of stroke, liver cirrhosis, anticoagulant or antiplatelet use, and blood-pressure control among hypertensive patients. Polyp- and procedure-related variables included polyp number, maximum polyp diameter, HRA, high-risk serrated polyp (HRSP), resection technique (verified manually), polyp morphology and location, prophylactic clipping, intraprocedural bleeding, endoscopist seniority (≥10 years senior vs. <10 years junior), bowel-preparation quality [Aronchick or Boston Bowel Preparation Scale (BBPS)], and sedation type (unsedated vs. sedated) (12, 13). Laboratory measures were platelet count and estimated glomerular filtration rate.

Periprocedural antithrombotic management followed institutional practice in consultation with the prescribing specialty: warfarin was typically interrupted 5 days before EMR, with the INR checked before the procedure and bridged with low-molecular-weight heparin in patients judged to have high thrombotic risk; non-vitamin K antagonist oral anticoagulants were typically interrupted at least 48 h pre-procedure based on renal function without bridging; antiplatelet agents were continued or interrupted according to individual thrombotic risk, as bridging is not applicable to antiplatelet therapy; agents were resumed after hemostasis was secured, with the timing individualized according to each patient's thrombotic and bleeding risk.

All EMR procedures followed a standardized electrosurgical protocol on an ERBE VIO unit (ERBE Elektromedizin, Tübingen, Germany): Endocut Q mode (blended cut–coagulation) with Effect 3, Duration 1, and Interval 6 (peak voltage 770 Vp) was used for resection, and forced coagulation at Effect 2 and 40 W (peak voltage 1,100 Vp) for hemostasis. Submucosal injection was performed with normal saline through a 23–25 G injection needle inserted obliquely into the submucosa at an angle of 30–45°, with the volume adjusted to lesion size until adequate lift-off was achieved. These settings remained unchanged throughout the study period.

HRA was defined as any adenoma ≥1 cm in diameter, harboring a villous component or high-grade dysplasia, or the presence of three or more adenomas. HRSP was defined as a serrated lesion that was either a sessile serrated polyp ≥1 cm or showed dysplasia (10). DPPB was defined as any bleeding event within 30 days after the procedure that prompted an outpatient or emergency visit or repeat endoscopic intervention. Events were identified through retrospective review of the hospital electronic record—captured from discharge diagnoses and readmission records throughout the 30-day window—without active patient follow-up, so admissions to other hospitals and minor self-limited bleeding may have been missed.

Prophylactic clipping was performed at the discretion of the operating endoscopist; no standardized institutional protocol governed clip placement during the study period. Clip status (any clip placed during the procedure) was ascertained from the free-text procedure reports (available for 4,004 of 4,035 procedures); lesion-level clip attribution and the number of clips were not uniformly documented and were not analyzed.

### 2.3 Statistical analysis

Continuous variables were expressed as mean ± standard deviation and compared with Student's *t*-test; categorical variables were summarized as frequencies and proportions, *n* (%), and compared using the χ^2^ test or Fisher's exact test. To assess the independent association of HRA with DPPB, we built three prespecified sequential (nested) logistic regression models: Model 1 included HRA alone (unadjusted); Model 2 additionally adjusted for age, sex, and BMI; Model 3 further adjusted for diabetes, hypertension, anticoagulant or antiplatelet use, and renal impairment (CKD stages 2–5). All covariates were entered by forced entry without stepwise selection based on univariable *P*-values. Where a covariate showed complete separation within a stratum (zero events in one cell), standard maximum-likelihood estimation becomes unbounded; in that setting we used Firth (1993) penalized likelihood regression, which yields finite estimates with profile-likelihood confidence intervals. Calendar year was assessed in a sensitivity adjustment to account for secular changes in endoscopic practice. To probe whether the inverse crude associations of diabetes and antithrombotic use with DPPB reflected differential process of care, prophylactic clip receipt (ascertainable in 4,004/4,035 procedures) was compared across comorbidity groups and additionally modeled as a binary outcome with lesion characteristics and comorbidities as predictors.

In the pathology-stratified analysis, patients were classified into four groups—neither HRA nor HRSP, HRA only, HRSP only, and coexisting HRA and HRSP—and both crude and adjusted odds ratios (ORs) were computed. Additive interaction indices (relative excess risk due to interaction [RERI], attributable proportion [AP], and synergy index [S]) and a multiplicative interaction term evaluated the joint effect of HRA and HRSP. For the size-stratified analysis (≤1 cm vs. > 1 cm), logistic regression adjusting for HRA and the covariates above—excluding polyp size and number—was fitted within each stratum to test whether the HRA effect varied with size, and a multiplicative interaction term (HRA × size category) assessed the significance of this modification. We assessed multicollinearity using variance inflation factors (VIF).

Variable selection was prospectively guided by clinical accessibility, prior literature, and univariable results, retaining five independent predictors: age (years), sex, maximum polyp diameter (cm), polyp count, and BMI (kg/m^2^). To corroborate that this clinically prespecified set was not an artifact of *post-hoc* selection, we additionally performed a Lasso (L1, 10-fold cross-validation) analysis over a twelve-candidate pool. Pathology (HRA) was deliberately omitted because the score is intended as a point-of-care intraprocedural tool, whereas HRA becomes available only after pathological review. Each variable was categorized at clinically meaningful cut-points: age as ≥60, 50–59, or <50; sex as female or male; maximum diameter as <1.0, 1.0–1.9, or ≥2.0 cm; polyp count as 1, 2–3, or ≥4; and BMI as ≥24 or <24 kg/m^2^.

Discrimination was quantified by the area under the receiver operating characteristic curve (AUC); the DeLong test compared the score with the full model. For internal validation we used a Harrell optimism bootstrap that rebuilds the entire score on every resample—multivariable refit, coefficient-to-integer rounding (B = 0.40), and score-model refit—and corrected both the AUC and the calibration slope. Calibration was appraised with the Hosmer–Lemeshow test, calibration intercept and slope, and the Brier score. Total scores stratified patients into low-, medium-, and high-risk groups at the empirical tertiles. All tests were two-sided, with *P* < 0.05 deemed significant. Analyses were performed in R, version 4.3.1 (R Foundation for Statistical Computing, Vienna, Austria).

## 3 Results

### 3.1 Patient selection and baseline characteristics

Within the analytic set, all twelve key variables (age, sex, BMI, maximum polyp diameter, polyp count, HRA, HRSP, platelet count, CKD stage, diabetes, hypertension, and anticoagulant/antiplatelet use) had 0.00% missing, so complete-case analysis could not introduce missing-data bias (Supplementary Table S6). Of 4,356 patients who underwent EMR during the study period, 4,035 were retained for analysis after excluding 68 with inflammatory bowel disease, 10 with familial polyposis, and 243 with incomplete records (Figure 1). DPPB occurred in 106 (2.63%), while the remaining 3,929 (97.37%) did not bleed. Baseline characteristics of the two groups are compared in Table 1. Patients who bled were younger (51.14 ± 12.76 vs. 56.81 ± 11.71 years, *P* < 0.001), more often male (78.30% vs. 62.10%, *P* < 0.001), and less likely to have diabetes (6.60% vs. 14.81%, *P* = 0.018) or to be taking anticoagulants or antiplatelets (5.66% vs. 12.14%, *P* = 0.043). Their polyps were also more numerous (≥6 lesions: 15.09% vs. 8.02%, *P* < 0.001), larger (maximum diameter 1.41 ± 0.58 vs. 1.11 ± 0.52 cm, *P* < 0.001), and more likely to be HRA (82.08% vs. 64.77%, *P* < 0.001) or HRSP (16.98% vs. 8.83%, *P* = 0.004). BMI, hypertension, CKD stage, platelet count, endoscopist seniority, bowel-preparation score, and sedation type did not differ between the groups.

**Table 1:** Baseline characteristics of study patients.

Variable	Total (*n* = 4,035)	Non-bleeding (*n* = 3,929)	Bleeding (*n* = 106)	*P* value
Age, mean ± SD	56.67 ± 11.77	56.81 ± 11.71	51.14 ± 12.76	<0.001
BMI, mean ± SD	25.14 ± 3.45	25.15 ± 3.45	24.77 ± 3.38	0.272
Gender, *n* (%)				<0.001
Male	2,523 (62.53)	2,440 (62.10)	83 (78.30)	
Female	1,512 (37.47)	1,489 (37.90)	23 (21.70)	
Hypertension, *n* (%)	1,416 (35.09)	1,380 (35.12)	36 (33.96)	0.805
Blood pressure control (among HTN), *n* (%)				0.428
Controlled	838 (59.18)	819 (59.35)	19 (52.78)	
Uncontrolled	578 (40.82)	561 (40.65)	17 (47.22)	
Diabetes mellitus, *n* (%)	589 (14.60)	582 (14.81)	7 (6.60)	0.018
Coronary heart disease, *n* (%)	255 (6.32)	251 (6.39)	4 (3.77)	0.275
History of cerebral infarction, *n* (%)	125 (3.10)	123 (3.13)	2 (1.89)	0.773
Liver cirrhosis, *n* (%)	16 (0.40)	15 (0.38)	1 (0.94)	0.347
CKD stage 2, *n* (%)	987 (24.46)	969 (24.66)	18 (16.98)	0.069
CKD stage 3–5, *n* (%)	127 (3.15)	126 (3.21)	1 (0.94)	0.262
Anticoagulant/antiplatelet use, *n* (%)	483 (11.97)	477 (12.14)	6 (5.66)	0.043
Platelet count, mean ± SD	228.40 ± 56.75	228.50 ± 56.66	224.81 ± 60.21	0.510
Number of polyps, *n* (%)				<0.001
<3	2,758 (68.35)	2,705 (68.85)	53 (50.00)	
3–5	946 (23.44)	909 (23.14)	37 (34.91)	
≥6	331 (8.20)	315 (8.02)	16 (15.09)	
Maximum polyp diameter (cm), mean ± SD	1.12 ± 0.52	1.11 ± 0.52	1.41 ± 0.58	<0.001
High-risk adenoma, *n* (%)	2,632 (65.23)	2,545 (64.77)	87 (82.08)	<0.001
High-risk serrated polyp, *n* (%)	365 (9.05)	347 (8.83)	18 (16.98)	0.004
Endoscopist experience, *n* (%)				0.823
Senior (≥10 years)	873 (21.64)	851 (21.66)	22 (20.75)	
Junior (<10 years)	3,162 (78.36)	3,078 (78.34)	84 (79.25)	
Bowel preparation scale, *n* (%)				0.560
Aronchick	1,787 (44.29)	1,743 (44.36)	44 (41.51)	
BBPS	2,248 (55.71)	2,186 (55.64)	62 (58.49)	
Aronchick score, *n* (%)				0.056
Score 1	160 (8.95)	156 (8.95)	4 (9.09)	
Score 2	947 (52.99)	919 (52.73)	28 (63.64)	
Score 3	592 (33.13)	585 (33.56)	7 (15.91)	
Score 4	85 (4.76)	80 (4.59)	5 (11.36)	
Score 5	3 (0.17)	3 (0.17)	0 (0.00)	
BBPS total score, mean ± SD	7.24 ± 1.27	7.24 ± 1.27	7.19 ± 1.20	0.786
Colonoscopy type, *n* (%)				0.408
Conventional	568 (14.08)	556 (14.15)	12 (11.32)	
Painless (sedated)	3,467 (85.92)	3,373 (85.85)	94 (88.68)	

Continuous variables: mean ± SD (Student's *t*-test). Categorical variables: *n* (%) (Chi–square test or Fisher's exact test).

CKD, chronic kidney disease; BMI, body mass index; BBPS, Boston Bowel Preparation Scale.

Among the 483 patients using antithrombotic agents, the agents comprised aspirin (*n* = 375, including 17 on dual antiplatelet therapy), P2Y12 inhibitors (*n* = 84), non-vitamin K antagonist oral anticoagulants (*n* = 14), warfarin (*n* = 5), and other agents (*n* = 23, predominantly indobufen and traditional Chinese medicines with antiplatelet activity), categories are not mutually exclusive. Notably, bridging-eligible patients (warfarin users) constituted only 0.12% of the cohort.

### 3.2 Univariable analysis

Univariable logistic regression (Table 2) linked DPPB to age (OR 0.96 per year, *P* < 0.001), male sex (OR 2.20, *P* < 0.001), polyp count (OR 1.10 per additional lesion, *P* < 0.001), maximum polyp diameter (OR 1.86 per cm, *P* < 0.001), HRA (OR 2.49, *P* < 0.001), and HRSP (OR 2.11, *P* = 0.005). Diabetes (OR 0.41, *P* = 0.022) and anticoagulant or antiplatelet use (OR 0.43, *P* = 0.049) emerged as protective in this unadjusted analysis, a pattern likely reflecting selection bias or clinical intervention. To test whether these inverse associations reflected differential process of care, we compared prophylactic clipping rates across comorbidity groups: clipping did not differ by diabetes (80.7% vs. 79.1%, *P* = 0.41) or antithrombotic use (79.5% vs. 79.3%, *P* = 0.98), and clip receipt was independently associated with lesion size >1 cm (aOR 2.82) and HRA (aOR 1.42) but with neither comorbidity (diabetes aOR 1.06, *P* = 0.60; antithrombotic use aOR 0.91, *P* = 0.45).

**Table 2:** Univariable logistic regression analysis for risk factors of delayed post-polypectomy bleeding.

Variable	OR (95% CI)	*P* value
Renal insufficiency (CKD 2–5)	0.57 (0.34–0.93)	0.026
Age (per year)	0.96 (0.95–0.98)	<0.001
Male sex	2.20 (1.38–3.51)	<0.001
BMI (per kg/m^2^)	0.97 (0.91–1.03)	0.272
Hypertension	0.95 (0.63–1.43)	0.805
Diabetes mellitus	0.41 (0.19–0.88)	0.022
Coronary heart disease	0.57 (0.21–1.57)	0.281
Cerebral infarction	0.60 (0.15–2.44)	0.471
Liver cirrhosis	2.49 (0.33–18.99)	0.380
Anticoagulant/antiplatelet use	0.43 (0.19–1.00)	0.049
Platelet (per 10^9^/L)	1.00 (1.00–1.00)	0.509
Polyp count (per 1)	1.10 (1.04–1.17)	<0.001
Maximum polyp size (per cm)	1.86 (1.48–2.32)	<0.001
High-risk adenoma	2.49 (1.51–4.11)	<0.001
High-risk serrated polyp	2.11 (1.26–3.55)	0.005
Junior endoscopist	1.06 (0.66–1.70)	0.823
Painless colonoscopy	1.29 (0.70–2.37)	0.410

CKD, chronic kidney disease; BMI, body mass index.

### 3.3 Independent association of HRA with DPPB: sequential adjustment models

Sequential multivariable logistic regression (Table 3) revealed a robust HRA–DPPB association across all three models: unadjusted Model 1 (OR 2.49, 95% CI 1.51–4.11, *P* < 0.001), Model 2 adjusting for age, sex, and BMI (OR 2.97, 95% CI 1.78–4.97, *P* < 0.001), and the fully adjusted Model 3 (OR 2.90, 95% CI 1.74–4.85, *P* < 0.001). Counterintuitively, the HRA odds ratio rose rather than fell after adjustment for age and other confounders (2.49 → 2.97).

**Table 3:** Univariable and sequential multivariable logistic regression analyses of factors associated with delayed post-polypectomy bleeding.

Variable	Univariable OR (95% CI)	Univariable P	Model 1 OR (95% CI)	Model 1 P	Model 2 OR (95% CI)	Model 2 P	Model 3 OR (95% CI)	Model 3 P
High-risk adenoma	2.49 (1.51–4.11)	<0.001	2.49 (1.51–4.11)	<0.001	2.97 (1.78–4.97)	<0.001	2.90 (1.74–4.85)	<0.001
Age (per year)	0.96 (0.95–0.98)	<0.001	—	—	0.95 (0.94–0.97)	<0.001	0.96 (0.94–0.97)	<0.001
Male sex	2.20 (1.38–3.51)	<0.001	—	—	2.14 (1.32–3.47)	0.002	2.16 (1.33–3.51)	0.002
BMI (per kg/m^2^)	0.97 (0.91–1.03)	0.272	—	—	0.94 (0.89–1.00)	0.038	0.93 (0.88–0.99)	0.025
Diabetes mellitus	0.41 (0.19–0.88)	0.022	—	—	—	—	0.52 (0.23–1.15)	0.104
Hypertension	0.95 (0.63–1.43)	0.805	—	—	—	—	1.51 (0.96–2.39)	0.077
Anticoagulant/antiplatelet	0.43 (0.19–1.00)	0.049	—	—	—	—	0.62 (0.26–1.46)	0.273
CKD 2–5	0.57 (0.34–0.93)	0.026	—	—	—	—	0.85 (0.50–1.46)	0.562

We constructed three sequential multivariable logistic regression models. Model 1 included only the primary variable of interest (HRA). Model 2 additionally adjusted for age, sex, and BMI. Model 3 further adjusted for diabetes mellitus, hypertension, anticoagulant/antiplatelet use, and CKD 2–5. Polyp size and count were not adjusted in primary models (see stratified analysis for natural control by size).

OR, odds ratio; CI, confidence interval; BMI, body mass index; CKD, chronic kidney disease.

Within Model 3, male sex was an additional independent risk factor (OR 2.16, *P* = 0.002), whereas age (OR 0.96 per year, *P* < 0.001) and BMI (OR 0.93 per kg/m^2^, *P* = 0.025) were protective. Adding polyp size and count to Model 3 attenuated the HRA estimate from 2.90 to 2.12 (95% CI 1.25–3.62, *P* = 0.006), which retained significance (Supplementary Table S1). No harmful collinearity was detected (HRA VIF 1.05 in the primary model and 1.23 after adding size and count; all variables < 2; Supplementary Table S5). Size and count therefore partly confound the HRA–DPPB link. This estimate sits alongside the component-decomposition result (3.02 → 1.71, *P* = 0.06; same table), in which size alone is added to a different covariate set; the two analyses bracket the HRA effect between a fully adjusted estimate that stays significant (2.12) and a size-only decomposition that does not (1.71), indicating that size accounts for a substantial share, though not all, of the association.

### 3.4 Pathology-stratified analysis: joint effects of HRA and HRSP

Pathology-stratified analysis (Table 4) took patients with neither HRA nor HRSP as the reference (bleeding rate 1.23%). HRA alone carried a rate of 3.04% (adjusted OR 2.90, 95% CI 1.66–5.07, *P* < 0.001), HRSP alone 2.97% (adjusted OR 2.27, 95% CI 0.64–8.01, *P* = 0.202), and coexisting HRA and HRSP the highest rate, 5.68% (adjusted OR 5.54, 95% CI 2.67–11.49, *P* < 0.001). Additive interaction testing yielded an adjusted RERI of 1.37 (95% CI −2.65 to 5.38), S of 1.43, and AP of 0.25 (Table 5). The intervals are wide and cross zero, in part because the HRSP-only group contained only three events, so these indices support a trend toward positive additive interaction rather than a statistically significant one; nominally, a quarter of the risk in the co-presence group is attributable to the interaction. On the multiplicative scale the interaction OR was 0.84 (95% CI 0.21–3.36, *P* = 0.805), indicating independent effects. Each high-risk pathology thus raised DPPB risk on its own, and the two together posed the greatest hazard (Supplementary Table S4).

**Table 4:** Pathology-stratified analysis of post-EMR bleeding risk.

Pathology group	n	Bleeding *n* (%)	Crude OR (95% CI)	Crude P	Adjusted OR* (95% CI)	Adjusted P*
Neither HRA nor HRSP	1,302	16 (1.23)	1.00	—	1.00	—
HRA only	2,368	72 (3.04)	2.52 (1.46–4.35)	<0.001	2.90 (1.66–5.07)	<0.001
HRSP only	101	3 (2.97)	2.46 (0.70–8.59)	0.158	2.27 (0.64–8.01)	0.202
Both HRA + HRSP	264	15 (5.68)	4.84 (2.36–9.92)	<0.001	5.54 (2.67–11.49)	<0.001

*Adjusted for age, sex, BMI, diabetes, hypertension, anticoagulant/antiplatelet use, and CKD stages 2–5 (not adjusted for polyp size or count).

**Table 5:** Interaction between HRA and HRSP.

Measure	Crude	Adjusted*
RERI (Relative Excess Risk due to Interaction)	0.86	1.37 (−2.65 to 5.38)
AP (Attributable Proportion due to Interaction)	0.18	0.25 (−0.39 to 0.89)
S (Synergy Index)	1.29	1.43 (−0.13 to 2.99)
Multiplicative interaction OR (95% CI)	—	0.84 (0.21–3.36)
Multiplicative interaction P	—	0.805

*Adjusted for age, sex, BMI, diabetes, hypertension, anticoagulant/antiplatelet use, and CKD 2–5 (not adjusted for polyp size/count).

RERI > 0 indicates positive additive interaction; AP > 0 indicates proportion attributable to interaction; S > 1 indicates synergism.

HRA, high-risk adenoma; HRSP, high-risk serrated polyp; RERI, relative excess risk due to interaction; AP, attributable proportion; S, synergy index.

### 3.5 Stratification by polyp size: effect modification of HRA

Size-stratified analysis (Table 6) disclosed a significant HRA × size interaction (interaction OR 0.22, 95% CI 0.08–0.63, *P* = 0.005). Among polyps ≤1 cm (*n* = 2,621; 38 bleeds), HRA multiplied DPPB risk 3.23-fold (OR 3.23, 95% CI 1.50–6.97, *P* = 0.003), whereas among polyps >1 cm (*n* = 1,414; 68 bleeds) no residual association was observed (OR 0.80, 95% CI 0.38–1.69, *P* = 0.561)—an estimate reflecting near-universal exposure in this stratum, since 1,269/1,414 patients (90%) were HRA-positive through the size criterion alone. Within the ≤1 cm stratum no diabetic patient bled, producing complete separation for diabetes under standard estimation; Firth penalized likelihood returned a finite estimate (OR 0.082, profile-likelihood CI 0.001–0.584, *P* = 0.065) (Supplementary Table S3), read as a selection artifact rather than a genuine protective effect of diabetes.

**Table 6:** Stratified analysis of post-EMR bleeding risk by polyp size.

Variable	Polyp ≤1 cm OR (95% CI)	P	Polyp >1 cm OR (95% CI)	P
High-risk adenoma	3.23 (1.50–6.97)	0.003	0.80 (0.38–1.69)	0.561
Age (per year)	0.97 (0.94–1.00)	0.083	0.95 (0.93–0.97)	<0.001
Male sex	1.77 (0.82–3.86)	0.148	2.78 (1.48–5.21)	0.001
BMI (per kg/m^2^)	0.98 (0.89–1.08)	0.692	0.91 (0.84–0.98)	0.018
Diabetes mellitus	0.082 (0.001–0.584)^†^	0.065	0.94 (0.40–2.18)	0.878
Hypertension	1.69 (0.81–3.54)	0.164	1.38 (0.76–2.49)	0.287
Anticoagulant/antiplatelet	0.30 (0.04–2.24)	0.240	0.72 (0.27–1.92)	0.506
Renal insufficiency (CKD 2–5)	0.84 (0.35–2.01)	0.688	0.84 (0.42–1.68)	0.622

HRA × Polyp Size Interaction: interaction OR = 0.22 (0.08–0.63), *P* = 0.005.

Polyp size and count were not included as covariates in stratified models (natural control within strata).

*N* = 2,621 (38 events) in ≤1 cm stratum; *N* = 1,414 (68 events) in >1 cm stratum.

† Estimated by Firth penalized likelihood with profile-likelihood confidence intervals (complete separation: no diabetic patient bled within the ≤1 cm stratum).

Because the HRA definition itself incorporates a≥10 mm criterion, the exposure and the stratifying threshold share the 1 cm boundary; we therefore repeated the analysis with this definitional overlap removed. Within the ≤1 cm stratum, lesions recorded as exactly 1.0 cm—the only subgroup that can simultaneously satisfy the HRA size criterion and remain classified as small—accounted for 1,175/2,621 patients (45%) and 26/38 bleeding events (68%), and recorded sizes showed strong digit preference (1.0 cm in 1,175 patients, vs. 33 at 0.9 cm and 6 at 1.1 cm), so the boundary group is best read as a rounding mixture of true 9–11 mm lesions. In a boundary-decomposition model within this stratum, an exactly-1.0 cm indicator entered together with a size-independent histology exposure (villous component, high-grade dysplasia, or ≥3 adenomas) assigned the apparent small-polyp effect to the boundary lesions themselves (adjusted OR 2.53, 95% CI 1.32–4.87, *P* = 0.005) rather than to histology (adjusted OR 1.52, 95% CI 0.78–2.94, *P* = 0.219). Under this non-size definition, the ≤1 cm estimate attenuated from 3.23 to 1.66 (95% CI 0.78–3.34, *P* = 0.136), the >1 cm stratum regained an informative contrast (adjusted OR 2.04, 95% CI 1.21–3.44, *P* = 0.008), and the exposure × size interaction disappeared (interaction OR 1.03, *P* = 0.95). Modelling size as a restricted cubic spline likewise showed no interaction for either the original HRA (joint *P* = 0.48) or the non-size definition (joint *P* = 0.85): histology predicted bleeding with a broadly stable adjusted OR of approximately 1.5–2.1 across 0.5–2.0 cm, whereas size itself showed a steep independent dose–response (vs. 0.8 cm: adjusted OR 1.65 at 1.0 cm, 2.55 at 1.2 cm, 4.35 at 1.5 cm, and 7.43 at 2.0 cm). The interaction observed in Table 6 is therefore definition-dependent rather than biological: size and histology each carry independent bleeding-risk information across the size spectrum.

Decomposing HRA into its components further examined the contribution of size to the HRA effect (Supplementary Table S1). Across the full cohort the HRA odds ratio fell from 3.0 to 1.71 (*P* = 0.06) once polyp size was added in the component-decomposition model, yet within ≤1 cm polyps HRA retained an independent effect (adjusted OR 3.21, 95% CI 1.48–6.98, *P* = 0.003), and a polyp count ≥3 predicted bleeding independently in both strata (≤1 cm adjusted OR 2.50; >1 cm 2.28). These decomposition estimates differ slightly from the main-analysis values (univariable HRA OR 2.49; size-stratified ≤1 cm HRA OR 3.23 in Table 6) because the component model adjusts for a different covariate set. The bleeding load of multiple polyps is therefore distinct from the histology captured by HRA. When intraprocedural variables extracted from the free-text reports were added to the model, pedunculated (Ip) morphology emerged as the independent predictor (adjusted OR 2.04, 95% CI 1.35–3.08, *P* = 0.0007), and prophylactic clipping carried an adjusted OR of 2.72 (95% CI 1.50–4.95)—a direction best explained by confounding by indication: in the absence of a protocolized clipping policy, clip placement tracked the endoscopist's real-time perception of bleeding risk (clip use 92.5% among patients who developed DPPB vs. 79.1% among those who did not; approximately 92% in the >1 cm stratum). Size >1 cm remained the dominant driver (adjusted OR 2.09) (Supplementary Table S2). Calendar year showed no significant temporal trend across 2016–2025 (adjusted OR 1.08 per year, *P* = 0.12; Supplementary Table S13).

### 3.6 Development and validation of the clinical risk score

The Lasso analysis over a twelve-candidate pool retained all twelve candidates at the optimal penalty (C = 3.92); the five clinically prespecified predictors were all retained (5/5 overlap), with age, polyp size, and polyp count entering the coefficient path first, corroborating that the clinically chosen set was not an artifact of *post-hoc* selection (Supplementary Table S7). The multivariable regression spanned eight non-reference categories across five predictors, with effect directions matching clinical expectation—younger age, male sex, larger diameter, higher polyp count, and lower BMI each raised bleeding risk. The strongest contributors were a maximum diameter ≥2.0 cm (OR 7.480, 95% CI 3.599–15.549), followed by 1.0–1.9 cm (OR 3.676, 95% CI 1.980–6.825) and age <50 years (OR 3.173, 95% CI 1.966–5.120). Following Sullivan and colleagues, we scaled the regression coefficients to integers with a constant B = 0.40, producing a 0–13-point score (Table 7); the largest single contribution came from a diameter ≥2.0 cm (5 points) and the smallest from age 50–59 years, 2–3 polyps, and BMI <24 kg/m^2^ (1 point each).

**Table 7:** Sullivan score assignment table for the 5-variable simplified risk score.

Variable	Category	β (logit)^a^	OR (95% CI)	W = β × (X_i − X_ref)^b^	Points^c^
Age (years)	≥60	0	1.00	0.0000	0
	50–59	0.4606	1.585 (0.944–2.660)	0.4606	1
	<50	1.1546	3.173 (1.966–5.120)	1.1546	3
Sex	Female	0	1.00	0.0000	0
	Male	0.7109	2.036 (1.258–3.294)	0.7109	2
Maximum polyp diameter (cm)	<1.0	0	1.00	0.0000	0
	1.0–1.9	1.3018	3.676 (1.980–6.825)	1.3018	3
	≥2.0	2.0123	7.480 (3.599–15.549)	2.0123	5
Polyp count (n)	1	0	1.00	0.0000	0
	2–3	0.3978	1.489 (0.908–2.442)	0.3978	1
	≥4	0.9935	2.701 (1.581–4.615)	0.9935	2
BMI (kg/m^2^)	≥24	0	1.00	0.0000	0
	<24	0.4444	1.560 (1.044–2.330)	0.4444	1
Intercept (β₀)	—	−6.3577	—	—	—

a β coefficients from the multivariable logistic regression on categorized predictors (reference categories set to β = 0).

b For categorical predictors, X_i = 1 for the index category and X_ref = 0 for the reference category; hence W_i = β_i. All W≥0, guaranteeing a non-negative integer score.

c Points = round(W_i/B), where B = 0.40 (e^0.40 ≈ 1.49; each 1-point increment ≈ OR 1.49 ≈ + 50% risk). Total score range = 0–13. Reference-group baseline risk = 0.17%. Risk formula: P(bleeding) = 1/(1 + e^(6.358 − 0.40 × Total Score)).

**Figure 2:**
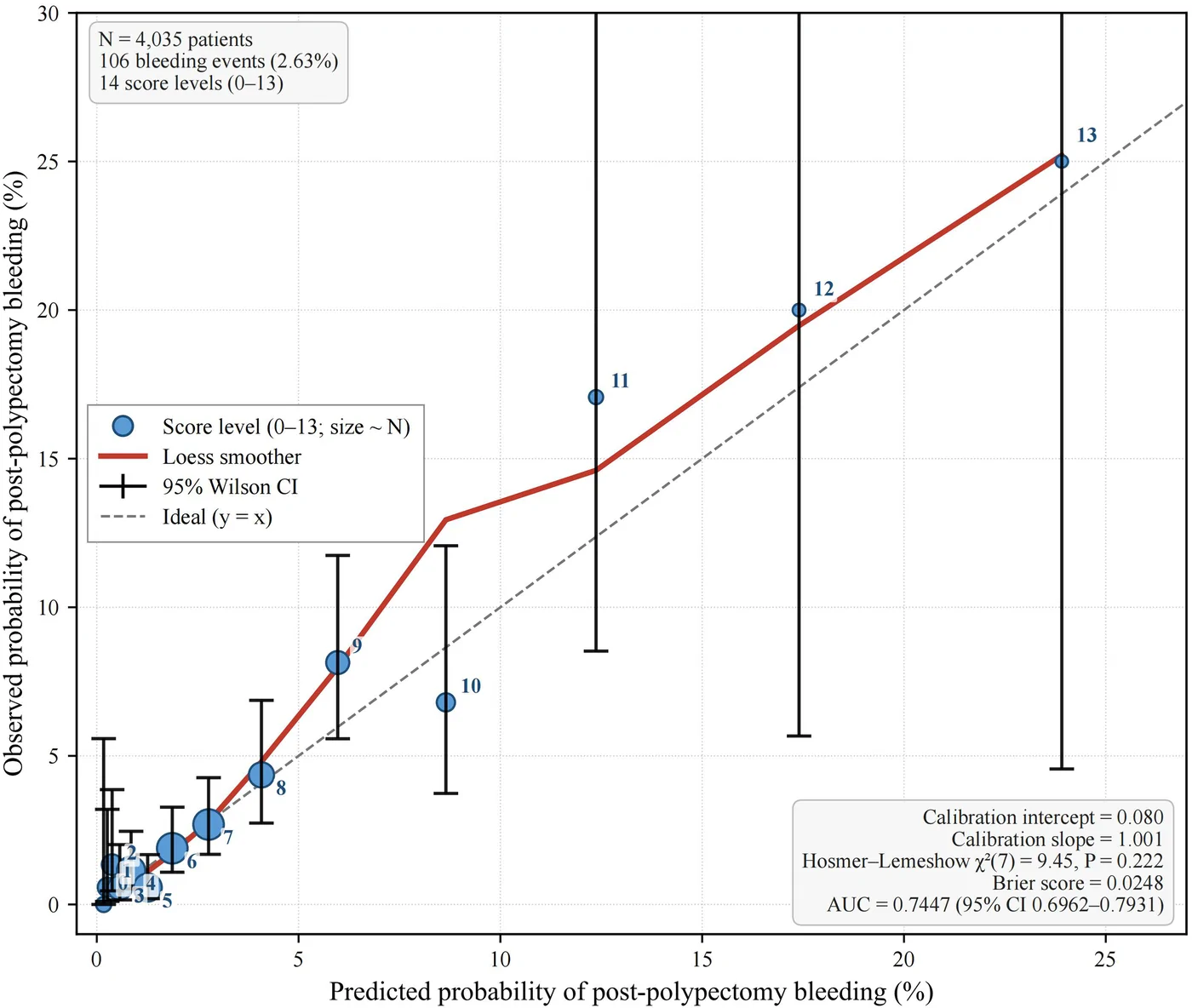
Calibration of the 5-variable simplified risk score. Blue circles represent observed bleeding probabilities at each score level (size proportional to N), with 95% Wilson confidence intervals shown as vertical bars. The red curve represents a Loess smoother. The dashed gray line indicates ideal calibration (y = x). Calibration intercept = 0.080; calibration slope = 1.001; Hosmer–Lemeshow χ^2^(7) = 9.45, *P* = 0.222; Brier score = 0.0248; AUC = 0.7447 (95% CI 0.6962–0.7931).

Patients were then grouped by total score into low (0–3), medium (4–8), and high (9–13) risk (Table 8). The low-risk group comprised 820 patients (20.3%) with 6 bleeds (0.73%); the medium-risk group 2,706 (67.1%) with 55 bleeds (2.03%); and the high-risk group 509 (12.6%) with 45 bleeds (8.84%). Although only 12.6% of the cohort, the high-risk group concentrated 42.5% of all bleeding events and carried roughly 12 times the risk of the low-risk group. Predicted bleeding rates tracked observed rates closely across score levels (Figure 2), with Wilson 95% confidence intervals largely overlapping the ideal diagonal.

**Table 8:** Risk stratification according to the simplified score (*N* = 4,035; 106 events).

Risk group	Score range	N	% of cohort	Bleeding events, n	Bleeding rate (%)
Low risk	0–3	820	20.3	6	0.73
Medium risk	4–8	2,706	67.1	55	2.03
High risk	9–13	509	12.6	45	8.84
Total	0–13	4,035	100.0	106	2.63

The high-risk group (12.6% of the cohort) accounted for 42.5% of all bleeding events, with an absolute rate approximately 12 × that of the low-risk group (8.84% vs. 0.73%). The medium-risk group captured 51.9% of events; the low-risk group captured 5.7%.

**Figure 3:**
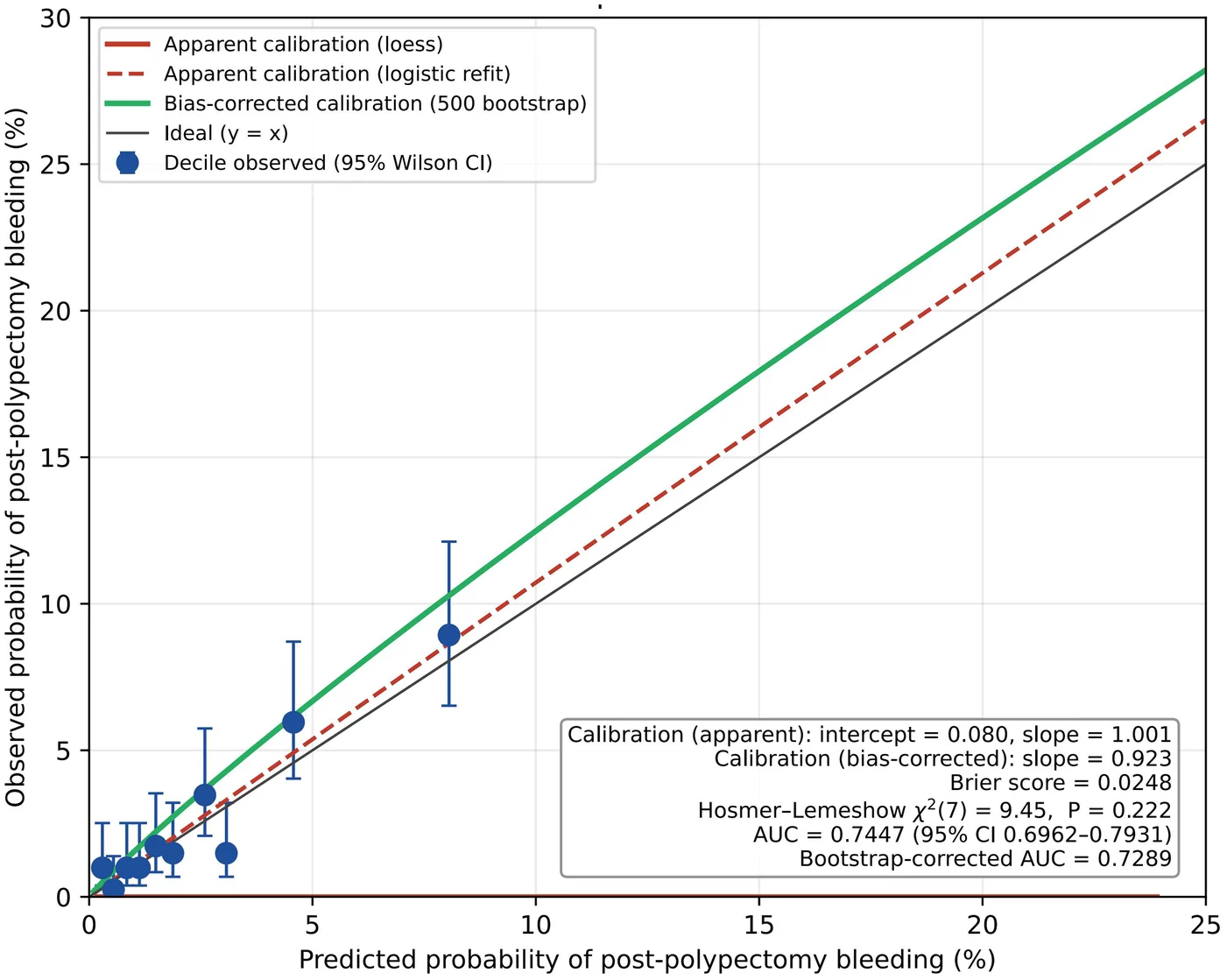
Calibration plot of the simplified risk score with bootstrap internal validation. Blue circles with error bars represent decile observed probabilities with 95% Wilson confidence intervals. The red solid curve and dashed logistic refit line depict apparent calibration. The green curve represents bias-corrected calibration (500 bootstrap resamples). The darkened reference line indicates ideal calibration (y = x). Bootstrap-corrected AUC = 0.7289.

**Figure 4:**
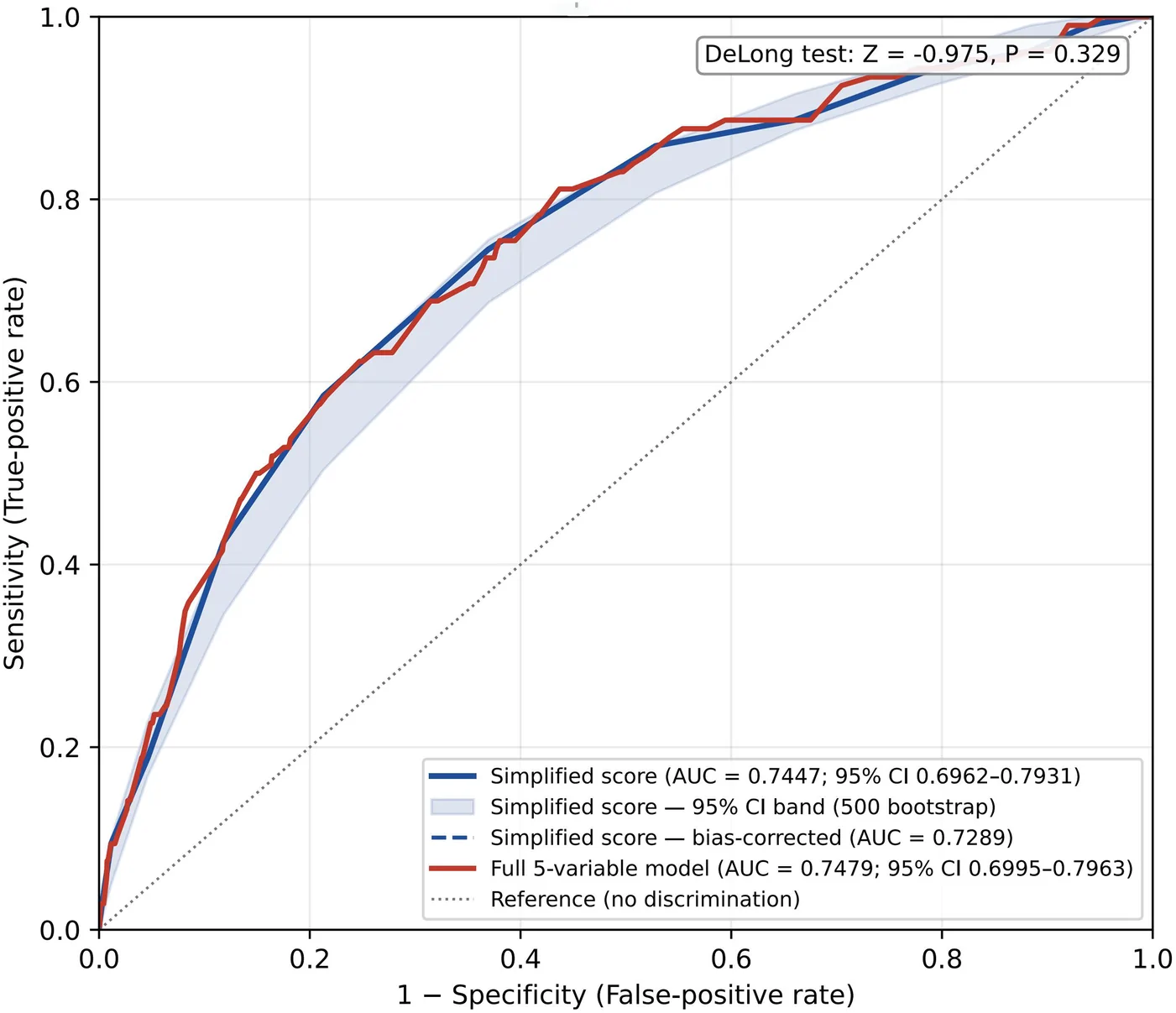
ROC curves of the simplified score vs. full model with bootstrap internal validation. The dark blue curve depicts the simplified score (AUC = 0.7447, 95% CI 0.6962–0.7931), with the shaded band representing 95% CI (500 bootstrap). The red curve depicts the full 5-variable model (AUC = 0.7479, 95% CI 0.6995–0.7963). The bias-corrected simplified score AUC is 0.7289 (dashed blue line). The gray diagonal line represents no discrimination. DeLong test: Z = −0.975, *P* = 0.329.

Discrimination and calibration of the simplified score and the full five-variable model are compared in Tables 8, 9 and Figures 3, 4. The AUC was 0.7447 (95% CI 0.6962–0.7931) for the score vs. 0.7479 (95% CI 0.6995–0.7963) for the full model, an insignificant difference (ΔAUC = −0.0032, DeLong *Z* = −0.975, *P* = 0.329). The optimism bootstrap, which rebuilt the score on every resample, returned a corrected AUC of 0.729 (optimism 0.016) and a corrected calibration slope of 0.923 (Supplementary Table S10). Calibration was otherwise satisfactory: Hosmer–Lemeshow χ^2^(7) = 9.445, *P* = 0.222; calibration intercept 0.080 and apparent slope 1.001; Brier score 0.0248. Reclassification gains were marginal (NRI 0.041, IDI 0.0026). The AIC values (simplified 899.35; full 911.94) are reported for completeness only (Supplementary Table S11); the two models are not meaningfully ranked by AIC, because the score is a rounded restriction of the full model and the apparent ΔAIC of 12.59 reflects the parameter-count penalty rather than a fit advantage (likelihood-ratio χ^2^ = 1.42, df = 7, *P* = 0.985).

**Table 9:** Performance metrics of the simplified score vs. the full 5-variable logistic regression model.

Domain	Metric	Simplified score (0–13)	Full 5-variable model^a^
Discrimination	AUC (C-statistic)	0.7447	0.7479
	95% CI	(0.6962–0.7931)	(0.6995–0.7963)
	Bootstrap-corrected AUC^b^	0.7289	—
	Optimism	0.0157	—
	DeLong test (Z; P)	—	−0.975; 0.329
Calibration	Hosmer–Lemeshow χ^2^ (df)	9.445 (7)	8.814 (8)
	H–L *P*-value	0.222	0.358
	Calibration intercept^c^	0.080	0.000
	Calibration slope^c^	1.001 (apparent)/0.923 (corrected)	1.000
	Brier score	0.0248	0.0248
Reclassification^d^	NRI (overall)	—	0.041
	NRI (events)	—	0.076
	NRI (non-events)	—	−0.034
	IDI	—	0.0026
Overall fit	AIC	899.35	911.94
	ΔAIC	—	12.59 (parameter-count penalty, not a fit advantage)
	No. of parameters (k)	2	9

Interpretation: ΔAUC = 0.003 (DeLong *P* = 0.329), comparable H–L *P* (>0.20), and identical Brier scores indicate the simplified score retains essentially all discriminative and calibrated performance of the full model while being more parsimonious (2 vs. 9 parameters). The AIC values are reported for completeness only; the two models are not meaningfully ranked by AIC, because the score is a rounded restriction of the full model and the apparent ΔAIC reflects the parameter-count penalty rather than a fit advantage (likelihood-ratio χ^2^ = 1.42, df = 7, *P* = 0.985).

a Full model = multivariable logistic regression with the same 5 categorized predictors.

b 500-resample bootstrap (Steyerberg/Harrell heuristic).

c Logistic refit of observed outcome on logit of predicted probability (ideal: intercept = 0, slope = 1).

d Reclassification metrics using the simplified score as the “old” model and the full regression as the “new” model, with a 5% risk-threshold boundary.

## 4 Discussion

Drawing on 4,035 EMR-treated patients, this study systematically appraised the HRA–DPPB relationship and arrived at three principal findings. First, HRA independently predicts DPPB (adjusted OR 2.90), and the association strengthened rather than weakened as major confounders were added sequentially, a descriptive pattern consistent with negative confounding by age (younger patients bled more often), offered without causal attribution to the adjustment itself. Second, size and histology carry independent, complementary risk information rather than interacting: non-size histology predicted bleeding across the size spectrum (adjusted OR ≈ 1.5–2.1, no effect modification), while polyp size itself exerted a steep, independent dose–response (up to OR 7.4 at 2 cm). Third, a score built from five simple variables discriminates DPPB risk effectively—a 12-fold gradient between high- and low-risk groups—and shows good discrimination and calibration after bootstrap validation.

The adenoma–DPPB link has seldom been examined; our three sequential models demonstrate that the HRA effect is stable (9, 14). The HRA odds ratio climbed from 2.49 to 2.97 once age was adjusted for, because younger patients in this cohort bled more often than older ones—a direction reported in several earlier series as well (15). This pattern reads more coherently as selection bias than as biology. Younger patients undergo colonoscopy for symptoms, a family history of neoplasia, or a known polyp rather than for screening, assembling a higher-risk subset (indication bias); at the other extreme, patients aged ≥80 who reach colonoscopy are disproportionately fit (healthy-survivor bias). Restricted cubic splines (four knots) detected no significant nonlinearity for age (*P* = 0.18) or BMI (*P* = 0.37), so the linear estimates are retained (Supplementary Table S12).

The stratified pattern—an elevated point estimate within ≤1 cm polyps (OR 3.23) and a null estimate beyond 1 cm (OR 0.80)—initially suggested that the HRA effect varies with polyp size, but four complementary analyses indicate that the pattern is definition-dependent rather than biological. The mechanism lies at the 1 cm boundary itself, the one point shared by the HRA definition and the stratification: lesions recorded as exactly 1.0 cm accounted for 45% of the ≤1 cm stratum (1,175/2,621) and 68% of its bleeding events (26/38), and recorded sizes cluster strongly at this value (1,175 patients at 1.0 cm vs. 33 at 0.9 cm and 6 at 1.1 cm), making the boundary group a rounding mixture of true 9–11 mm lesions. Within the ≤1 cm stratum, entering the exactly-1.0 cm indicator and a size-independent histology exposure in the same model assigned the apparent small-polyp effect to the boundary lesions themselves (adjusted OR 2.53, 95% CI 1.32–4.87, *P* = 0.005) rather than to histology (adjusted OR 1.52, 95% CI 0.78–2.94, *P* = 0.219). The null estimate beyond 1 cm, in turn, reflected exposure saturation—1,269/1,414 patients (90%) were HRA-positive through the size criterion alone, leaving only 145 referents—rather than irrelevance of histology, which regained an informative contrast under the non-size definition (adjusted OR 2.04, 95% CI 1.21–3.44, *P* = 0.008); the interaction vanished under either remedy (non-size exposure, *P* = 0.95; continuous size modelling, *P* = 0.48–0.85). Consistent with size and histology carrying independent information, the HRA odds ratio falls from 3.0 to 1.71 (*P* = 0.06) once size is added in the component-decomposition model across the full cohort, while a polyp count ≥3 predicts bleeding independently in both strata (≤1 cm adjusted OR 2.50; >1 cm 2.28) (Supplementary Table S1). The sensitivity analysis restricted to lesions strictly smaller than 1.0 cm still rests on 12 events, with point estimates in the same direction under either definition (original HRA OR 3.05; non-size definition 2.96) but profile CIs including 1 (Supplementary Table S14), so a histology signal in truly diminutive lesions can be neither confirmed nor excluded. The interaction is therefore best read as a definitional artifact; its practical message survives in modified form—small polyps do not carry uniformly low bleeding risk, chiefly because lesions at the 1 cm boundary bleed far more often than smaller ones (2.2% vs. 0.83%), with at most a suggestive histology contribution below 1.0 cm (16).

Pathology stratification showed the highest DPPB risk when HRA and HRSP coexisted (adjusted OR 5.54; bleeding rate 5.68%), with adjusted additive indices of RERI 1.37 (95% CI −2.65 to 5.38), S 1.43, and AP 0.25. These values are consistent with a trend toward positive additive interaction, although the wide intervals—driven by the three events in the HRSP-only group—temper the strength of the conclusion; nominally, a quarter of the risk in the co-presence group is attributable to the interaction. The multiplicative term was non-significant (OR 0.84, *P* = 0.805), showing no departure from independent multiplicative effects. Even if the two factors act through biologically separate channels, their combination can push absolute bleeding risk beyond the sum of the individual effects. HRA may heighten bleeding because villous adenomas carry richer microvascular structure, and high-grade dysplasia entails architectural disarray and neovascularization, both of which leave the post-EMR wound prone to oozing or arterial hemorrhage (17). HRSP may operate differently: serrated lesions often grow flat or laterally and cluster in the right colon, a region where several studies record higher post-polypectomy bleeding rates (18).

The multiplicative test asks whether the joint effect exceeds the product of the individual effects, whereas the additive test asks whether the absolute risk difference surpasses the sum of the individual risk differences. Here the multiplicative interaction OR of 0.84 (*P* = 0.805) indicates the joint effect stays within the range expected of a product, hinting that HRA and HRSP may partly overlap in their causal pathways. The additive indices (RERI 1.37, AP 0.25), though estimated on sparse events, nominally place 25% of the excess absolute risk among patients exposed to both high-risk polyp types on synergy between them. Reference patients (neither HRA nor HRSP) bled at 1.23% vs. 5.68% in the co-presence group, an absolute difference of 4.45 percentage points; per 1,000 co-presence patients roughly 57 bleed vs. 12 in the reference group, leaving about 45 excess events attributable to the dual exposure. The standalone HRSP estimate rests on sparse events (3 bleeds among 101 patients), but this does not weaken the highly significant co-presence effect (*P* < 0.001). Patients carrying both high-risk adenoma and high-risk serrated features are, in absolute terms, a very-high-risk subgroup for DPPB; whether measures such as prophylactic hemostatic clipping, extended post-procedural observation, or closer outpatient follow-up (19) should be adopted for them is a consideration to be tested prospectively, not a recommendation supported by this single-center retrospective association.

BMI emerged as protective against DPPB (OR 0.93 per kg/m^2^, *P* = 0.025), each unit lowering bleeding risk by 7%. Such a direction has seldom been reported and merits discussion, though the effect is modest: the 7% reduction per unit carries a upper confidence limit (0.99), so its clinical import may be limited. The inverse association can still be read in three ways.

The first is an obesity paradox: across critical illnesses such as heart failure, end-stage renal disease, and respiratory failure, overweight or obese patients often fare better (20). This pattern is ascribed to a subclinical procoagulant state (20). In this state, higher BMI tracks with elevated fibrinogen, heightened platelet activity, and inhibited fibrinolysis—a thrombotic tilt that, in a bleeding context, may act protectively (21).

The second concerns nutritional reserve and tissue repair: patients with higher BMI typically carry greater reserves and may synthesize protein and regenerate tissue more readily, so the post-polypectomy wound heals faster and the bleeding window narrows (22). The third is operator behavior: larger patients are often presumed to be technically harder, prompting endoscopists toward more aggressive prevention—more prophylactic clips, longer intraprocedural observation (23). The BMI–DPPB link is therefore unlikely to reflect a single mechanism but rather the combined action of coagulation, reserve, and operator bias, a question prospective studies should resolve.

The inverse pattern extends beyond age and BMI. Diabetes mellitus, renal impairment, and antithrombotic use each trended protective in the unadjusted analysis (OR 0.41, 0.57, and 0.43, respectively) yet attenuated toward the null under multivariable adjustment (Model 3: diabetes OR 0.52, *P* = 0.104; anticoagulant or antiplatelet use OR 0.62, *P* = 0.273; CKD stages 2–5 OR 0.85, *P* = 0.562). These inverse crude associations most plausibly reflect a combination of patient- and lesion-case-mix confounding and unmeasured periprocedural risk mitigation—chiefly antithrombotic agent interruption—rather than a biological shield conferred by the comorbidity itself; differential prophylactic clipping was specifically excluded as a contributing channel, since clipping rates did not differ by diabetes (80.7% vs. 79.1%) or antithrombotic use (79.5% vs. 79.3%), and clip receipt was driven by lesion risk rather than comorbidity. Consistent with risk-adapted management, antithrombotic-treated patients underwent resection of larger lesions (mean maximum diameter 1.21 vs. 1.11 cm, *P* < 0.001) yet developed DPPB less often (5.66% of bleeders vs. 12.14% of non-bleeders, *P* = 0.043). Each association is read descriptively, without causal attribution.

The simplified score offers clear practical advantages. It rests on only five variables—age, sex, BMI, polyp size, and polyp count—each available before or during the procedure, so the endoscopist can stratify risk and decide on prophylaxis before leaving the table, turning risk assessment into a real-time bedside judgment. Pathology (high-risk adenoma) was deliberately left out because it is unavailable until after the procedure; a Lasso analysis over a twelve-candidate pool retained all twelve candidates at the optimal penalty, with the five clinically chosen predictors all among them and age, size, and count entering the coefficient path first; adding HRA to the score left discrimination unchanged (ΔAUC 0.000; Supplementary Table S8), confirming that its information is already captured by size and count. The score also dispenses with complex comorbidity or lifestyle data. Earlier predictive models have incorporated diabetes, hypertension, anticoagulation, smoking, and alcohol intake, information that demands detailed history-taking and electronic-record review, inflating the time cost of data collection and degrading completeness and accuracy through reliance on patient recall and chart quality (11, 24). The five variables chosen here are objective, readily captured, and immune to subjective reporting bias, and they require no prior records, reducing the chance of missing data. The score needs no device or online tool: an integer point table that any clinician can use, with zero training barrier and strong portability.

The three-tier stratification is intended to inform rather than dictate management, and its boundaries are robust rather than arbitrary: the integer cuts track the empirical 33rd and 67th score percentiles, and sensitivity analyses across median split, quartile, and a 5% risk-threshold rule produced comparable gradients (high-to-low rate ratios 4.8–12.1), so the layering does not hinge on a single cutpoint (Supplementary Table S9). The gradient itself is descriptive: low-risk patients (0.73%) bled in fewer than 1 in 100 cases, whereas high-risk patients (8.84%) bled at roughly 12 times the low-group rate. How such risk information might eventually guide decisions on prophylaxis, observation, resection strategy, scheduling, or follow-up is a matter for prospective multicenter study; the present single-center retrospective association does not warrant prescribing routine prophylactic clipping, extended observation, staged resection, or day-case discharge for any risk tier.

Several limitations warrant mention. First, the retrospective design cannot rule out unmeasured confounding; intraprocedural variables (polyp morphology, prophylactic clipping, intraprocedural bleeding) were extracted from the free-text reports and entered the adjusted models. Moreover, prophylactic clipping was discretionary and unprotocolized, precluding adjustment for a structured clipping indication. En-bloc vs. piecemeal technique, defect size, antithrombotic interruption or resumption, the duration of post-procedure observation or inpatient admission, and periprocedural glycemic control were not captured in structured fields and remain unmeasured confounders. Second, DPPB was ascertained through retrospective review of the hospital electronic record without active 30-day patient contact, so admissions to other hospitals and minor self-limited bleeding may have been missed, and precise time-to-bleed was not uniformly recorded. Third, the analysis is patient-level: in patients with multiple lesions the largest diameter, total count, and HRA/HRSP status are summarized per patient without attributing the bleed to a specific lesion, which limits lesion-level inference. Fourth, the 106 bleeding events constrain power in subgroup analyses—only 3 events occurred in the HRSP-only group, and some covariates in the ≤1 cm stratum were estimated imprecisely, so those results call for cautious interpretation. Fifth, the score has undergone only internal bootstrap validation; its discrimination (AUC ≈ 0.73) is moderate, the optimism-corrected calibration slope is 0.923, and its generalizability is untested in an external cohort, which multicenter prospective studies will need to establish. Sixth, because the HRA definition embeds size (≥1 cm) and adenoma count (≥3), the HRA × size interaction must be interpreted with methodological care: a lesion of exactly 1.0 cm satisfies the HRA size criterion yet falls within the ≤1 cm stratum, so the ≤1 cm HRA estimate is largely carried by these boundary lesions. Further analyses showed that this interaction is definition-dependent—it disappeared when the size criterion was removed from the exposure (*P* = 0.95) or when size was modelled continuously (*P* = 0.48–0.85)—and that recorded sizes cluster at the 1.0 cm boundary (1,175 patients vs. 33 at 0.9 cm and 6 at 1.1 cm), a digit preference that makes the boundary group itself a rounding mixture of true 9–11 mm lesions. Within-stratum comparison was intended to control size by design, but the shared boundary leaves stratification itself vulnerable to this overlap, so continuous modelling of size is the more robust approach; restricting the ≤1 cm stratum to lesions strictly smaller than 1.0 cm left only 12 bleeding events and an HRA point estimate (OR 3.05) that was no longer significant, so any histology signal in truly diminutive lesions remains suggestive rather than conclusive. Seventh, calendar year was included as a covariate and showed no significant temporal trend across 2016–2025 (adjusted OR 1.08 per year, *P* = 0.12; Supplementary Table S13), yet unmeasured secular changes in practice may persist. Eighth, this is a single-center study, and external generalizability remains to be confirmed.

In summary, HRA is an independent risk factor for DPPB after EMR, and high-risk histology predicts bleeding across the size spectrum, while polyp size is itself a strong, independent risk factor. Polyp pathology should therefore enter bleeding-risk assessment on equal footing with size. A simplified five-variable score showed adequate discrimination and calibration on internal validation but, as a single-center instrument, awaits external confirmation.

## Data Availability

The original contributions presented in the study are included in the article/Supplementary Material, further inquiries can be directed to the corresponding author.
