# Supplementary material for: High-risk adenoma predicts delayed post-polypectomy bleeding: a size-stratified retrospective cohort study and risk score development
Source: Front Med (Lausanne). 2026 Aug 31;13:1913120. doi: 10.3389/fmed.2026.1913120 (PMC13572221; doi:10.3389/fmed.2026.1913120)
Supplement: Supplementary file 1 [file Table1.docx]

**Supplementary Material**

High-Risk Adenoma Predicts Delayed Post-Polypectomy Bleeding: A Size-Stratified Retrospective Cohort Study and Risk Score Development

Manuscript ID: 1913120 — Frontiers in Medicine (Gastroenterology)

This document provides the supplementary analyses referenced in the Response to Reviewers. Each table addresses one or more reviewer comments and uses the confirmed analytic dataset (N = 4,035; 106 DPPB events). All headline numbers reproduce Tables 1–8 of the main manuscript.

**Supplementary Table S1. HRA decomposed into components — size-stratified adjusted odds ratios (Reviewer 1, Comments 2 & 3)**

| **Stratum** | **HRA adjOR (95% CI)** | **p** | **Polyp count ≥3 vs 1, adjOR (95% CI)** | **p** | **Villous, OR (p)** | **HGD, OR (p)** |
| --- | --- | --- | --- | --- | --- | --- |
| ≤1 cm (n=2,621; 38 bleeds) | 3.21 (1.48–6.98) | 0.003 | 2.50 (1.11–5.64) | 0.027 | 2.18 (0.156) | 1.68 (0.625) |
| >1 cm (n=1,414; 68 bleeds) | 0.77 (0.36–1.63) | 0.49 | 2.28 (1.22–4.27) | 0.010 | — | — |

Adjusted for age, sex, hypertension, diabetes, anticoagulant use, and polyp count. Full-cohort model: adding polyp size attenuated HRA from 3.02 to 1.71 (0.98–2.98), p=0.060; polyp size >1 cm adjOR 2.94 (1.90–4.55), p<0.001 — the dominant driver. In the primary multivariable model, adding both polyp size and count attenuated HRA from 2.90 to 2.12 (1.25–3.62), p=0.006. (This size estimate (2.94) differs from the intraprocedural model in Supplementary Table S2 (2.09) because the two models adjust for different covariate sets.) Villous component and HGD did not reach significance within strata.

**Supplementary Table S2. Intraprocedural variables as independent predictors of DPPB (Reviewer 1, Comment 5)**

| **Variable** | **Adjusted OR (95% CI)** | **p** | **Interpretation** |
| --- | --- | --- | --- |
| Pedunculated (Ip) morphology | 2.04 (1.35–3.08) | 0.0007 | Strongest independent predictor |
| Prophylactic clipping | 2.72 (1.50–4.95) | 0.001 | Indication bias (high-risk lesions clipped) |
| Polyp size >1 cm | 2.09 (1.34–3.26) | 0.001 | Dominant lesion-level driver |
| Male sex | 2.22 (1.27–3.88) | 0.005 | — |
| Hypertension | 1.51 (1.00–2.29) | 0.050 | — |
| Age (per year) | 0.96 (0.94–0.98) | <0.001 | Inverse (selection; see S12) |

Adjusted for demographics and mutually for the listed intraprocedural variables. Complete intraprocedural data were available for 80 of 106 bleeding events (75.5%); the 26 missing events are concentrated in the earlier years of the cohort. Clipping OR is interpreted as indication bias, not a treatment effect.

**Supplementary Table S3. Firth penalized logistic regression for the ≤1 cm stratum (Reviewer 1, Comment 8)**

| **Variable** | **Firth OR** | **Wald 95% CI** | **Profile-likelihood 95% CI** | **p** |
| --- | --- | --- | --- | --- |
| High-risk adenoma | 3.10 | 1.51–6.39 | 1.52–6.94 | 0.002 |
| Age (per year) | 0.97 | 0.94–1.00 | 0.94–1.00 | 0.069 |
| Male sex | 1.70 | 0.82–3.54 | 0.82–3.84 | 0.154 |
| BMI (per kg/m²) | 0.98 | 0.90–1.08 | 0.89–1.08 | 0.699 |
| Diabetes mellitus | 0.082 | 0.006–1.17 | 0.001–0.584 | 0.065 |
| Hypertension | 1.70 | 0.84–3.42 | 0.81–3.51 | 0.138 |
| Anticoagulant/antiplatelet | 0.44 | 0.09–2.13 | 0.05–1.73 | 0.304 |
| Renal impairment (CKD 2–5) | 0.88 | 0.39–1.99 | 0.35–1.97 | 0.760 |

n = 2,621; 38 bleeding events. Standard MLE returned Diabetes OR = 0.00 (0.00–Inf) because 379 diabetic patients in this stratum had zero bleeds (complete separation). Firth (1993) penalized likelihood (logL + ½ log|I(β)|) yields finite estimates with profile-likelihood CIs, the recommended approach under separation. HRA OR under Firth penalization (3.10) is close to the standard estimate (3.23), confirming the headline is robust.

**Supplementary Table S4. Additive interaction between HRA and HRSP with delta-method 95% CIs (Reviewer 1, Comment 9)**

*S4a. Four-group table*

| **Pathology group** | **n** | **Bleeds** | **Crude OR (95% CI)** |
| --- | --- | --- | --- |
| Neither HRA nor HRSP (reference) | 1,302 | 16 | 1.00 |
| HRSP only | 101 | 3 | 2.46 (0.71–8.59) |
| HRA only | 2,368 | 72 | 2.52 (1.46–4.35) |
| Both HRA + HRSP | 264 | 15 | 4.84 (2.36–9.92) |

*S4b. Interaction indices*

| **Measure** | **Crude** | **Adjusted (95% CI)** |
| --- | --- | --- |
| RERI | 0.86 | 1.37 (−2.65 to 5.38) |
| AP | 0.18 | 0.25 (−0.39 to 0.89) |
| S | 1.29 | 1.43 (−0.13 to 2.99) |
| Multiplicative OR | — | 0.84 (0.21–3.36), p = 0.805 |

*Adjusted for age, sex, BMI, diabetes, hypertension, anticoagulant/antiplatelet use, and CKD 2–5 (not polyp size/count). All 95% CIs cross zero; the HRSP-only group contains only 3 events. The interaction is therefore reported as a non-significant trend; the prior causal/“25% attributable” framing was removed.*

**Supplementary Table S5. Collinearity diagnostics — variance inflation factors (Reviewer 1, Comments 2 & 3)**

| **Variable** | **VIF (primary model)** | **VIF (with size + count)** |
| --- | --- | --- |
| High-risk adenoma | 1.05 | 1.23 |
| Age | 1.42 | 1.44 |
| Male sex | 1.06 | 1.07 |
| BMI | 1.09 | 1.09 |
| Diabetes | 1.08 | 1.08 |
| Hypertension | 1.24 | 1.24 |
| Anticoagulant/antiplatelet | 1.13 | 1.13 |
| CKD 2–5 | 1.21 | 1.21 |
| Polyp size | — | 1.15 |
| Polyp count | — | 1.10 |

*Conventional threshold for harmful collinearity is VIF > 5. All values are < 2, confirming that the structural overlap of HRA with size/count does not produce harmful collinearity; the attenuation of HRA upon mutual adjustment reflects confounding/mediation, not numerical instability.*

**Supplementary Table S6. Missingness of key variables within the analytic set, N = 4,035 (Reviewer 1, Comment 7)**

| **Variable** | **Missing, n (%)** |
| --- | --- |
| Age | 0 (0.00) |
| Sex | 0 (0.00) |
| BMI | 0 (0.00) |
| Maximum polyp diameter | 0 (0.00) |
| Polyp count | 0 (0.00) |
| High-risk adenoma | 0 (0.00) |
| High-risk serrated polyp | 0 (0.00) |
| Platelet count | 0 (0.00) |
| CKD stage | 0 (0.00) |
| Diabetes | 0 (0.00) |
| Hypertension | 0 (0.00) |
| Anticoagulant/antiplatelet use | 0 (0.00) |

*All twelve key variables were complete in the analytic set, so complete-case analysis cannot introduce missing-data bias. The only coverage gap concerns the free-text intraprocedural fields (available for 80/106 bleeding events).*

**Supplementary Table S7. Lasso (L1) variable selection — data-driven retention (Reviewer 1, Comment 10)**

| **Candidate variable** | **Standardized coefficient** | **Retained** |
| --- | --- | --- |
| Age | −0.565 | Yes |
| BMI | −0.210 | Yes |
| Maximum polyp size | 0.289 | Yes |
| Polyp count | 0.182 | Yes |
| Platelet count | −0.137 | Yes |
| Male sex | 0.678 | Yes |
| Diabetes | −0.622 | Yes |
| Hypertension | 0.368 | Yes |
| Anticoagulant | −0.589 | Yes |
| CKD 2–5 | −0.182 | Yes |
| High-risk adenoma | 0.736 | Yes |
| High-risk serrated polyp | 0.455 | Yes |

*L1 penalty, 10-fold cross-validation, best C = 3.92. Coefficient path: age, polyp size, and polyp count enter first as the penalty increases. The five clinically prespecified predictors (age, sex, size, count, BMI) are all retained, corroborating that the score’s predictor set is not an arbitrary post-hoc selection.*

**Supplementary Table S8. Score with vs without HRA — discrimination comparison (Reviewer 1, Comment 10)**

| **Metric** | **5-variable score** | **6-variable score (+HRA)** |
| --- | --- | --- |
| AUC | 0.7447 | 0.7447 |
| ΔAUC (gain from HRA) | — | 0.000 (bootstrap 95% CI −0.004 to 0.009) |
| Sullivan integer points | 3/1, 2, 3/5, 1/2, 1 | identical; HRA point = 0 |
| Redundancy (HRA ~ size+count) | — | Nagelkerke R² = 0.271 |

*Adding HRA to the score does not improve discrimination and its integer weight rounds to zero, because HRA is largely redundant with the size and count variables already inside the score. Pathology is also unavailable intraoperatively, which is why the bedside score excludes it by design.*

**Supplementary Table S9. Risk-group cutpoint sensitivity analysis (Reviewer 1, Comment 11)**

*S9a. Alternative grouping strategies*

| **Strategy** | **Groups** | **High-group % cohort** | **High rate (%)** | **Low rate (%)** | **Rate ratio** |
| --- | --- | --- | --- | --- | --- |
| Current tertile (0–3/4–8/9–13) | 3 | 12.6 | 8.84 | 0.73 | 12.1 |
| Median split | 2 | 37.9 | 5.16 | 1.08 | 4.8 |
| Empirical tertile | 3 | 22.3 | 6.90 | 0.89 | 7.7 |
| Quartile | 4 | 22.3 | 6.90 | 0.89 | 7.7 |
| Risk-threshold (≥5%) | 3 | 12.6 | 8.84 | 1.08 | 8.2 |

*S9b. Binary operating characteristics*

| **Cutpoint (score ≥)** | **Sensitivity (%)** | **Specificity (%)** | **PPV (%)** |
| --- | --- | --- | --- |
| ≥7 | 74.5 | 63.0 | 5.16 |
| ≥8 | 58.5 | 78.7 | 6.90 |
| ≥9 | 42.5 | 88.2 | 8.84 |
| ≥10 | 18.9 | 95.4 | 9.90 |

*Gradients are robust across strategies (rate ratios 4.8–12.1), so stratification is not an artifact of one cutpoint. The integer cuts at 3|4 and 8|9 align with the empirical 33rd and 67th score percentiles (4 and 7).*

**Supplementary Table S10. Full Harrell optimism bootstrap, B = 500 (Reviewer 1, Comment 12)**

| **Metric** | **Apparent** | **Optimism** | **Optimism-corrected** |
| --- | --- | --- | --- |
| AUC | 0.7447 | 0.0157 | 0.7289 |
| Calibration slope | 1.0000 | 0.0773 | 0.9227 |

*Each resample rebuilds the entire score: multivariable logistic refit → coefficient-to-integer rounding (B = 0.40) → score-model refit, with optimism estimated for both AUC and calibration slope. The corrected calibration slope (0.923) is newly reported; previously only the apparent slope (1.001) was given.*

**Supplementary Table S11. Model-comparison audit — simplified score vs full 5-variable model (Reviewer 1, Comment 13)**

| **Metric** | **Simplified score (k = 2)** | **Full model (k = 9)** |
| --- | --- | --- |
| Log-likelihood | −447.68 | −446.97 |
| Deviance (−2ll) | 895.35 | 893.94 |
| AIC | 899.35 | 911.94 |
| AUC (apparent) | 0.7447 | 0.7479 |

*ΔAIC = deviance excess of the score (1.42) − parameter penalty saved (14) = −12.58. Likelihood-ratio test χ² = 1.42, df = 7, P = 0.985: the full model’s seven extra parameters buy no significant fit advantage, and discrimination is statistically identical (ΔAUC 0.003, DeLong P = 0.329). The score’s advantage is parsimony, not fit; the “better fit” claim was removed.*

**Supplementary Table S12. Restricted cubic splines and age-stratum pattern (Reviewer 1, Comment 14)**

*S12a. Nonlinearity test (4-knot RCS)*

| **Variable** | **Linear OR (95% CI)** | **Nonlinearity p** |
| --- | --- | --- |
| Age (per year) | 0.957 (0.940–0.975) | 0.18 |
| BMI (per kg/m²) | 0.941 (0.883–1.002) | 0.37 |

*S12b. Age-stratum pattern underlying the inverse association*

| **Age group** | **Bleeding rate (%)** | **Mean polyp count** | **Mean size (cm)** | **HRA (%)** | **>1 cm-stratum rate (%)** |
| --- | --- | --- | --- | --- | --- |
| <40 | 6.07 | 1.91 | 1.08 | 49 | 12.59 |
| 50–59 | 2.46 | 2.48 | 1.12 | 65 | 4.29 |
| ≥80 | 0.00 | 3.38 | 1.31 | 81 | 0.00 |

*No significant nonlinearity (P > 0.05), so linear estimates are retained. The inverse age association is reframed as indication bias (younger patients referred for symptoms/family history) and healthy-survivor bias (the very old who reach colonoscopy are disproportionately fit), replacing the earlier speculative peristalsis mechanism.*

**Supplementary Table S13. DPPB rate by calendar year and temporal trend (Reviewer 1, Comment 5)**

| **Year** | **n** | **Bleeds** | **Rate (%)** |
| --- | --- | --- | --- |
| 2016 | 59 | 0 | 0.00 |
| 2017 | 149 | 5 | 3.36 |
| 2018 | 281 | 6 | 2.14 |
| 2019 | 420 | 11 | 2.62 |
| 2020 | 221 | 8 | 3.62 |
| 2021 | 643 | 13 | 2.02 |
| 2022 | 676 | 19 | 2.81 |
| 2023 | 922 | 14 | 1.52 |
| 2024 | 432 | 16 | 3.70 |
| 2025* | 232 | 14 | 6.03 |

**2025 is a partial year (to 31 March). Unadjusted year OR = 1.069/year (P = 0.153); adjusted OR = 1.078/year (P = 0.121). No significant temporal trend in DPPB across 2016–2025; the HRA estimate was unchanged when calendar year was included as a covariate.*

**Supplementary Table S14. Sensitivity analysis — ≤1.0 cm vs <1.0 cm stratum definition (Reviewer 1, Comment 2)**

| **Stratum definition** | **n** | **Bleeds** | **HRA OR (95% CI)** | **p** |
| --- | --- | --- | --- | --- |
| ≤1.0 cm (main analysis) | 2,621 | 38 | 3.23 (1.50–6.97) | 0.003 |
| <1.0 cm (excludes 1.0-cm boundary) | 1,446 | 12 | 3.05 (0.88–10.48) | 0.077 |
| Exactly 1.0 cm | 1,175 | 26 | — | — |

*A lesion of exactly 1.0 cm satisfies the HRA ≥1 cm size criterion yet is classified within the ≤1 cm stratum; 26 of the 38 bleeds (68%) in the ≤1 cm stratum occur at exactly 1.0 cm. Excluding the boundary preserves the direction of the HRA effect but renders it non-significant (only 12 events remain). The small-polyp HRA signal is therefore suggestive rather than conclusive; this limitation is stated in the manuscript.*
